# Functional reconstitution of plant plasma membrane H^+^-ATPase into giant unilamellar vesicles

**DOI:** 10.1038/s41598-025-92663-9

**Published:** 2025-03-12

**Authors:** Huriye D. Uzun, Ekaterina Malysenko, Bo H. Justesen, Thomas Günther Pomorski

**Affiliations:** 1 Department of Molecular Biochemistry, Faculty of Chemistry and Biochemistry, Ruhr University Bochum, Bochum, Germany; 2 Department of Plant and Environmental Sciences, University of Copenhagen, Frederiksberg, Denmark

**Keywords:** Giant unilamellar vesicles, Membrane transporter, P-type ATPase, Electroformation, Gel-assisted swelling method, Charge-mediated fusion, Biochemistry, Biological techniques, Biophysics, Biotechnology, Plant sciences, Chemistry, Nanoscience and technology

## Abstract

Membrane transporters are essential for numerous biological processes by controlling the movement of ions and molecules across cell membranes. However, dissecting their molecular dynamics in complex cellular environments presents significant challenges. Reconstitution of membrane transporters in model systems offers a powerful solution. In this study, we focused on the reconstitution conditions suitable for the P3 ATPase *Arabidopsis thaliana* H^+^-ATPase isoform 2 and compatible with various giant unilamellar vesicle generation techniques. Among the methods evaluated for GUV formation, including electroformation, gel-assisted formation, and charge-mediated fusion, only the gel-assisted approach successfully generated AHA2-containing giant unilamellar vesicles while preserving the pump activity. Our findings underscore the importance of carefully managing the reconstitution conditions, including the presence of ions, and selecting the appropriate lipid composition to enhance the stability and activity of AHA2 in proteoliposomes. Addressing these factors is essential for the successful formation and functional analysis of AHA2 and other P-type ATPases in experimental settings.

**Supplementary Information:**

The online version contains supplementary material available at 10.1038/s41598-025-92663-9.

## Introduction

Membrane transporters play a critical role in many biological processes by facilitating the transport of ions and molecules across cell membranes. However, studying these membrane proteins and their molecular regulation poses significant challenges due to the complex cellular environment. Reconstitution in liposomal model membrane systems addresses these challenges by enabling studies under well-defined chemical conditions and allowing detailed biochemical, biophysical, and single-molecule analyses^1^. This approach has been particularly valuable in studying membrane transporters such as *Arabidopsis thaliana* H^+^-ATPase isoform 2 (AHA2), a member of the P3 subfamily of P-type ATPases. These essential proton pumps use ATP hydrolysis to translocate protons across the plasma membrane, thereby establishing and maintaining an electrochemical proton gradient. This gradient drives essential biological processes such as nutrient uptake, intracellular pH regulation, stomatal opening, and cell growth^2^.

Functional analysis of isolated H^+^-ATPases is typically performed by reconstitution into small (< 100 nm) or large (> 100 nm) unilamellar vesicles (SUVs or LUVs), which allow proton accumulation within the vesicle lumen^3–5^and studies down to the single vesicle level^6^. These proton pump assays have been instrumental in measuring the regulatory effect of lipids^7^on AHA2 function and in dissecting the role of specific protein domains and amino acids in the transport mechanism^8–10^. In addition, the coupling between ATP hydrolysis and proton transport rate for H^+^-ATPases can only be accurately analyzed within reconstituted proteoliposome systems^3,11^.

In this work, we aimed to reconstitute AHA2 into giant unilamellar vesicles (GUVs)^12–14^, which offer several advantages over SUVs and LUVs for studying transporters. With diameters ranging from 5 to 100 μm, GUVs are comparable in size to eukaryotic cells, enabling direct observation of transporter activity using conventional light microscopy^15–20^. The ability to visualize lipid domains in GUVs facilitates studies of lateral lipid heterogeneity and its impact on transporter behavior^21,22^. GUVs are also compatible with techniques such as micropipette aspiration and optical tweezers, allowing precise control of membrane tension, shape and curvature - factors critical to transporter function^23,24^. Furthermore, GUVs allow studies of the mechanical interplay between transporter activity and bending elasticity^25,26^.

Reconstitution of membrane transporters into GUVs is inherently challenging, as the conditions required for stable GUV formation often conflict with those required for functional protein incorporation. Several methods have been developed to address this issue, including electroformation^23,25,27–31^, spontaneous swelling^16,32–35^, membrane fusion of preformed protein-free GUVs with small proteoliposomes^36–38^, and detergent-mediated reconstitution^20,39^. All these methods have previously been used to reconstitute different P-type ATPases belonging to the P1 and P2 ATPase subfamilies (Suppl. Table S1), although using markedly different conditions and with only a few successful cases reported. Notably, the reconstitution of P3 ATPases into GUVs has not been documented to date.

In the present study, we focused on optimizing the reconstitution of active AHA2 into GUVs. Initially, we investigated how reducing ion concentrations in the reconstitution buffer and incorporating charged lipids for charge-mediated fusion influenced the proton-pumping activity of AHA2 in proteoliposomes. Subsequently, we explored three reconstitution techniques—electroformation, gel-assisted swelling, and charge-mediated fusion—to incorporate AHA2 into GUVs. To increase the proton transport rate, we used a 73 amino acid C-terminal truncated version of AHA2 that lacks parts of the autoinhibitory R-domain^5,40,41^, which is known to tightly regulate AHA2 activity^40–42^. This truncation results in a constitutively active enzyme, bypassing regulatory inhibition^43^.

## Results and discussion

Our strategy follows a two-step procedure, starting with the formation of large unilamellar proteoliposomes as starting material for the generation of protein-containing GUVs. The advantage of this two-step method is that the protein functionality in the proteoliposomes can be tested prior to GUV formation. This pre-evaluation step allows for optimization of ionic conditions, lipid composition, and reconstitution parameters, ensuring that only functional proteins are carried forward into the GUV formation stage.

### Buffer requirements and the effect of lipids on the functional reconstitution of AHA2

**Fig. 1:**
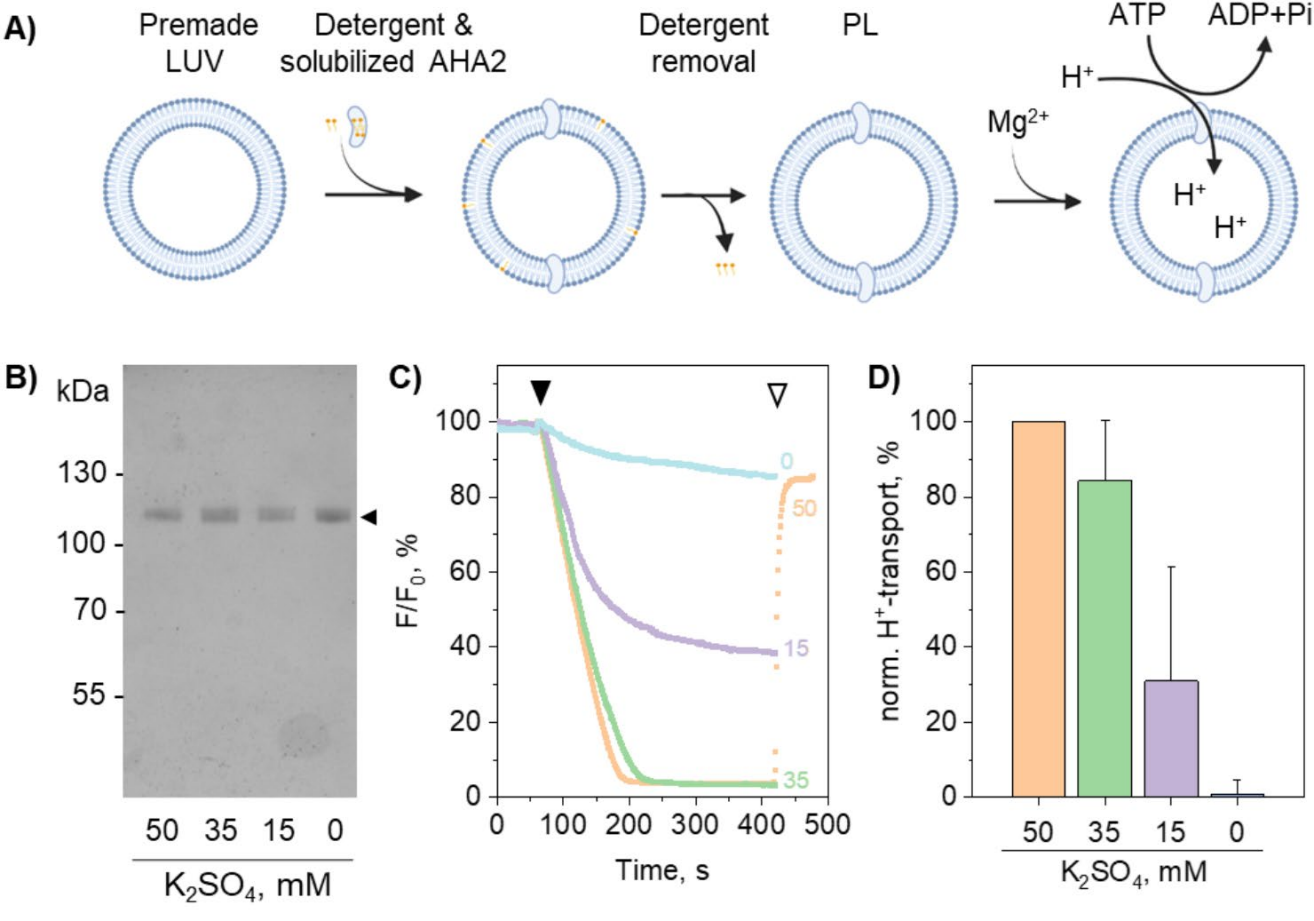
AHA2 activity upon reconstitution into large unilamellar vesicles at different ionic strengths. **A**) Schematic representation of the reconstitution based on preformed LUVs destabilized with detergent and proton transport assay. The addition of Mg^2+^ to ATP-containing buffer initiated ATP hydrolysis and proton pumping into the vesicle lumen. **B**) Coomassie Blue-stained SDS-PAGE gel of AHA2 reconstituted in proteoliposomes (PL) at the indicated ion concentrations. **C**) Proton transport into vesicles reconstituted with AHA2 was measured following ACMA fluorescence at different ionic strengths as indicated. ATP-stimulated proton pumping was initiated upon the addition of MgSO_4_ (filled arrowhead), and the proton gradient was collapsed by adding m-chlorophenylhydrazon (CCCP; open arrowhead). Traces are representative of three independent experiments. **D**) Proton transport activity of AHA2 was determined as initial rates of the fluorescence quenching of pH sensor ACMA, normalized to liposomal protein content, and expressed relative to the control (50 mM K_2_SO_4_, *n* = 3). Data represents three independent reconstitutions with two to four measurements each, shown as mean ± S.D. A value of 100% corresponds to 5.2 ± 1.9 × 10^−5^%/s.

Many GUV formation protocols are limited to low salt buffers and non-charged lipids. However, numerous studies have shown that specific cations and lipid composition significantly impact the activity of P-type ATPases^7,44–51^. Therefore, we first investigated the effect of these factors on the stability of AHA2 during the reconstitution process into preformed LUVs. Purified AHA2 was reconstituted into preformed liposomes using buffers with progressively decreasing K_2_SO_4_ concentrations (Fig. 1A, Suppl. Figure S1). SDS-PAGE analysis of the proteoliposome samples showed a single band corresponding to the expected size of AHA2 (~ 119 kDa), irrespective of the buffer composition (Fig. 1B, Suppl. Figure S1). Protein recovery after reconstitution was 62.8 ± 11.6% (*n* = 2; Suppl. Figure S1). The importance of maintaining a minimum K^+^ concentration during reconstitution is highlighted by the findings related to AHA2 proton pumping activity. When reconstituted in buffers of decreasing K^+^ concentration, AHA2 proteoliposomes showed a decrease in ATP-driven proton pumping, even though all proteoliposomes were resuspended in ACMA buffer containing 52.5 mM K_2_SO_4_ (Fig. 1C + D). Conceivably, monovalent cations protect P-type ATPases from inactivation, as previously observed for AHA2^52^ and Na^+^/K^+^-ATPase^51^. To preserve the activity of AHA2 during the reconstitution process, we chose to include 50 mM K_2_SO_4_ in the reconstitution buffers, although this condition is not ideal for the subsequent preparation of GUVs.

**Fig. 2:**
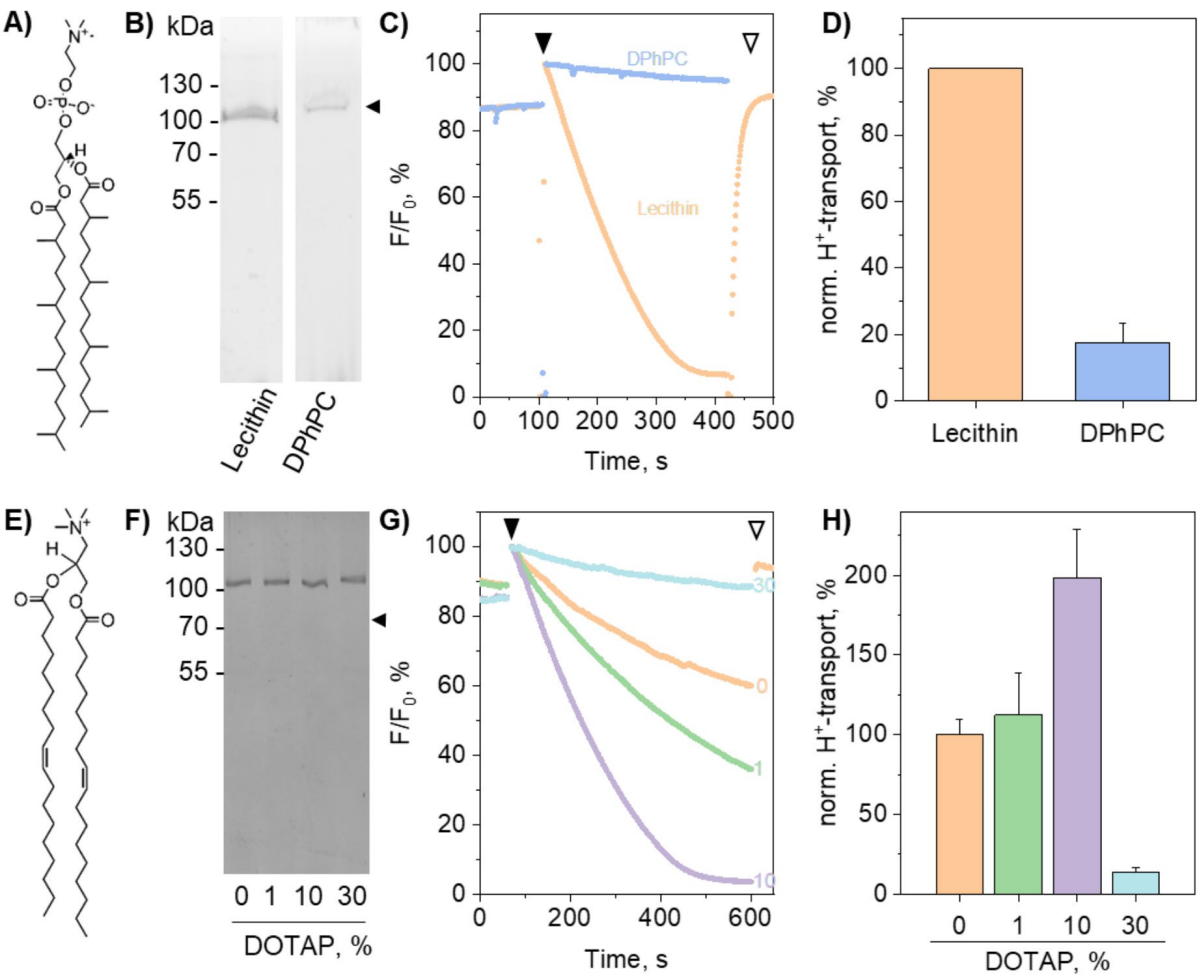
AHA2 activity upon reconstitution in LUVs containing special lipids. A, E) Structures of the lipids DPhPC (A) and DOTAP (E). B, F) Coomassie Blue-stained SDS-PAGE gel of AHA2 reconstituted in proteoliposomes containing the indicated lipids. C, G) Proton transport into vesicles reconstituted with AHA2 with indicated lipid compositions. Mg-ATP stimulated proton pumping was initiated upon the addition of MgSO_4_ (filled arrowhead), and the proton gradient was collapsed by adding CCCP (open arrowhead). The traces are representative of two independent experiments (panel C) and one experiment (panel G). D, H) Proton transport activity of AHA2 was determined as initial rates of the fluorescence quenching of the pH sensor ACMA, normalized to liposomal protein content, and expressed relative to the control. In panel D, data represents two independent reconstitutions with one measurement each, shown as mean ± S.D. A value of 100% corresponds to 5.6 ± 0.6 × 10^−5^%/s. In panel H, data represents one reconstitution with three measurements, shown as mean ± S.D. A value of 100% corresponds to 1.5 ± 0.2 × 10^−5^%/s. A second independent experiment is shown in Suppl. Figure S2.

We next investigated the effects of 1,2-diphytanoyl-*sn*-glycero-3-phosphocholine (DPhPC) and 1,2-dioleoyl-3-trimethylammonium-propane (DOTAP) lipids on AHA2 reconstitution and activity (Fig. 2A + E). DPhPC lipids have been shown to improve liposome integrity^15–17,34,39,53–55^, whereas DOTAP, a cationic lipid, is frequently used for charge-mediated vesicle fusion^36,37,56^. Including DPhPC in the reconstitution process resulted in a reduction in reconstitution efficiency (Fig. 2B) and was less effective in supporting proton pumping activity compared to reconstitutions without DPhPC (Fig. 2C + D). Conversely, reconstituting AHA2 into liposomes containing 1,2-dioleoyl-*sn*-glycero-3-phosphocholine (DOPC) and DOTAP led to the successful integration of AHA2, regardless of the DOTAP concentration (Fig. 2F, Suppl. Figure S2). However, the DOTAP concentration was critical for the proton pump activity; at 1% and 10% DOTAP, AHA2 remained active, whereas at 30% DOTAP hardly any proton pumping activity was observed (Fig. 2G + H, Suppl. Figure S2). Evidently, the functionality of the protein is strongly influenced by the liposomal lipid composition^7,57–60^, which therefore needs to be tested and selected carefully.

### Generation of giant vesicles with AHA2

**Fig. 3:**
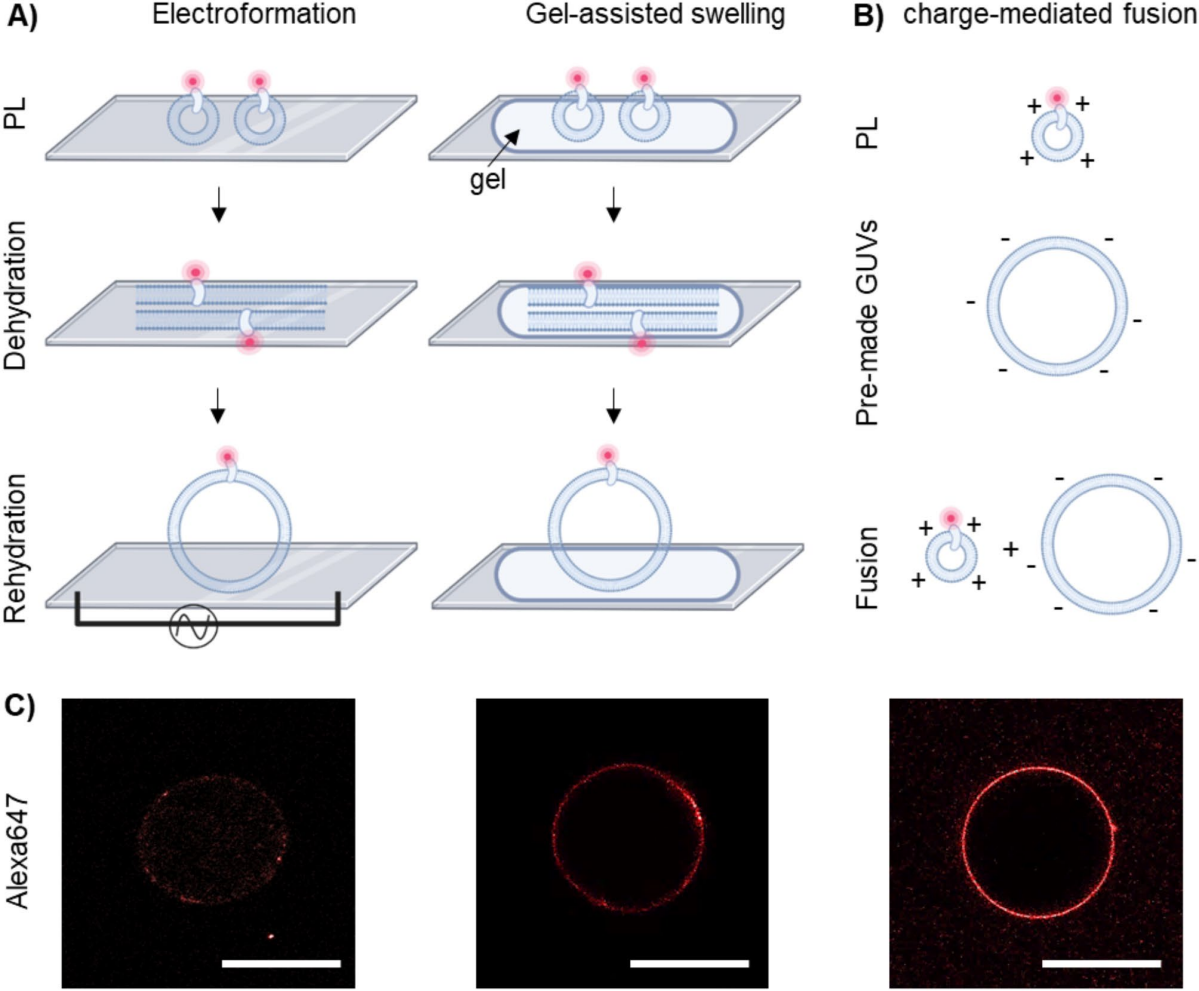
Generation of proteo-GUVs with AHA2. A, B) Schematic diagrams illustrating the formation of proteo-GUVs using different methods. **A**) Electroformation involves the rehydration of partially dehydrated proteoliposomes in the presence of an AC electric field. Gel-assisted swelling is achieved by rehydrating partially dehydrated proteoliposomes on a gel-coated glass slide. **B**) Charge-mediated fusion involves combining positively charged proteoliposomes with pre-formed negatively charged GUVs, driving fusion by electrostatic interactions. **C**) Confocal images showing fluorescence channels corresponding to Alexa647-labelled AHA2. Only electroformation and gel-assisted swelling yielded proteo-GUVs. In charge-mediated fusion, Alexa647 fluorescence was localized to adhering PLs rather than protein integrated into the GUV membranes (for details see Suppl. FigureS4). Scale bar, 20 μm.

Having established the buffer requirements for maintaining AHA2 activity during the reconstitution process and having investigated its tolerance to DPhPC and DOTAP lipids, we next tested three common approaches for functional reconstitution of membrane proteins into GUVs. To track AHA2 during the reconstitution process and to facilitate the identification of protein-containing vesicles, we used SNAP-tagged AHA2, which allows stoichiometric labelling with a fluorophore of choice via SNAP-tag technology^61^. Initial experiments showed that the proton pumping activity of Alexa647-labelled AHA2 was comparable to that of the unlabelled pump (Suppl. FigureS3). GUV formation based on electroformation and gel-assisted swelling generated AHA2-containing GUVs, albeit with different efficiencies (Fig. 3A + C). Electroformation, using settings similar to Garten et al. (2015) at 0.35 V and 500 Hz^15^, yielded only a few protein-containing GUVs (proteo-GUV; *n*= 3 ± 3, 10 experiments), with 93% of the GUVs containing AHA2. This limited yield might be attributed to the presence of ions within the proteoliposomes, which can prevent the proper dehydration required for GUV electroformation. In addition, the frequency of 500 Hz during rehydration might have been insufficient for optimal GUV generation. In the presence of high charge density in the swelling buffer and the liposomes, electroformation performs better at around 1 kHz and with higher voltage^62,63^. However, a higher electric field could induce lipid oxidation^64^. In our case, electroformation was not suitable for further studies, mainly due to the low number of proteo-GUVs generated. In contrast, the gel-assisted formation technique significantly increased the number of proteo-GUVs (*n* = 20 ± 10, 8 experiments), all containing AHA2.

Charge-mediated fusion was tested using proteoliposomes composed of DOPC:DOTAP (molar ratio 9:1) and pre-formed GUVs composed of DOPC and 1,2-dioleoyl-*sn*-glycero-3-phospho-(1’-rac-glycerol) (DOPG; in molar ratio 7:3) and resulted in AHA2 localization at the membrane (Fig. 3B + C). To assess the feasibility of LUV fusion with GUVs, LUVs composed of DOPC:DOTAP (molar ratio 9:1 or 7:3) and loaded with the luminal dye pyranine were incubated with pre-formed GUVs composed of DOPC:DOPG (molar ratio 7:3). In this setup, pyranine acted as a fusion marker that would diffuse into the GUV lumen upon successful fusion. However, pyranine fluorescence remained localized in the same area as the membrane marker in the GUVs, indicating that LUVs were adhering to the GUV surface rather than undergoing fusion (Suppl. Figure S4). Previous studies suggested that the inclusion of 1,2-dioleoyl-*sn*-glycero-3-phosphoethanolamine (DOPE) in LUVs can enhance the fusion process^65,66^. Under the conditions used in this study, we did still not observe fusion even when using LUVs containing DOPC:DOPE:DOTAP (molar ratio 6:3:1;Suppl. Figure S4). Conceivably, the presence of anions and cations in the buffer affects the fusion process, as noted in previous studies^65,67^.

### Proton pump activity in giant vesicles

**Fig. 4:**
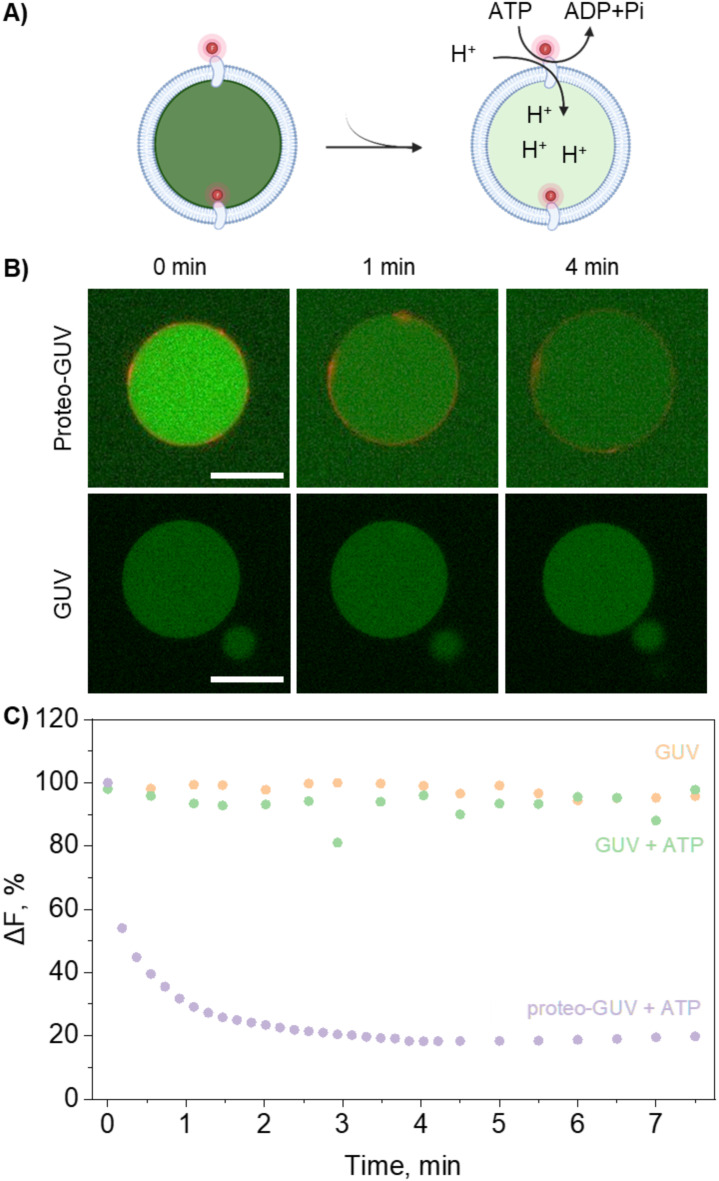
AHA2 activity upon reconstitution into proteo-GUVs. **A**) Illustration of Alexa647-labelled AHA2 reconstituted into proteo-GUVs and imaged on an individual GUV basis with confocal fluorescence microscopy. Extravesicular addition of both ATP and Mg^2+^ activated exclusively outward-facing AHA2, triggering proton pumping into the vesicle lumen. Changes in the vesicular proton concentration were monitored via the pH-sensitive fluorophore pyranine. Valinomycin was always present to mediate K^+^/H^+^ exchange and to prevent the build-up of a transmembrane electrical potential. **B**) Confocal images of proteo-GUVs and GUVs (control) formed via the gel-assisted swelling method in the presence of pyranine. Vesicles were analyzed by fluorescence microscopy before and after the addition of ATP. Scale bar, 20 μm. **C**) Pyranine fluorescence of proteo-GUVs and GUVs (control) measured as a function of time, normalized to the maximum fluorescence value. Additional activity traces are shown in Suppl. Figure S6.

To assess the proton transport activity of AHA2-containing GUVs generated by gel-assisted formation, we used the luminal pH sensor pyranine^39,68^. Here, unlike for the fusion control experiment described before, pyranine was already present in the GUVs and enabled tracking of pH variations within GUVs, indicating proton pumping activity by AHA2 in the presence of valinomycin and Mg-ATP (Fig. 4A). To validate this assay, we reconstituted AHA2 into preformed LUVs loaded with pyranine, confirming that fluorescence intensity changes corresponded to proton transport (Suppl. Figure S5). Pyranine fluorescence intensity exhibits a wide linear response to changes in proton concentration, with decreased fluorescence correlating with lower pH values, allowing direct interpretation of the area-corrected fluorescence intensity as an indication of proton pump-mediated proton transport^69,70^. Pyranine-loaded protein-free GUVs served as control in these experiments to exclude indirect effects by ATP addition. In the absence of ATP, pyranine fluorescence in individual AHA2-containing GUVs remained constant over the duration of the experiment, indicating no passive diffusion of the pH sensor pyranine from the GUVs or bleaching of the fluorophore (Fig. 4B + C). Conversely, in the presence of MgATP, the fluorescence intensity decreased to 20%, demonstrating ATP-driven proton transport by the proton pump in GUVs (Fig. 4B + C, Suppl. Figure S6).

Notably, out of 27 proteo-GUVs analysed, 5 showed ATP-induced proton pumping, representing 18% of the tested population across 10 preparations. This relatively low success rate may be attributed to the dehydration step during GUV formation, which has been reported to adversely affect the activity of membrane proteins, as observed in previous studies with a Ca^2+^-ATPase^23^. This could also account for the variability observed in the activity traces of the individual proteo-GUVs. Various strategies, such as the addition of sucrose, trehalose, or ethylene glycol during dehydration, have been shown to be effective in preserving protein activity during GUV formation^27,71^. However, these approaches can inhibit GUV formation^27^. Alternatively, dehydration via a controlled N_2_flow has been introduced to achieve rapid dehydration without vacuum^15^, but this approach was not successful in our hands.

## Conclusion

The generation of proteo-GUVs containing AHA2 is a task of balancing divergent requirements regarding buffer conditions and lipid composition for protein stability and GUV formation, as summarized in Suppl. Table S2. Most existing GUV formation protocols were incompatible with the potassium ion concentrations required for AHA2 functionality in liposomes. In addition, AHA2 was inactive in certain lipid compositions, such as those with high DOTAP concentrations, which may have hindered proteo-GUV formation via charge-mediated fusion. Of the methods tested (electroformation, gel-assisted formation, and charge-mediated fusion), only the gel-assisted approach generated GUVs with active AHA2 in sufficient yields. The successful generation of active AHA2 proteo-GUVs necessitated a medium salt buffer, as confirmed by the detection of proton transport activity using the pH-sensitive probe pyranine. The ability to generate proteo-GUVs with functional AHA2 provides a novel platform for the detailed characterization of this membrane transporter using techniques that are uniquely suited to GUVs, including flickering analysis and domain partitioning. Other membrane transporters might benefit from different proteo-GUV formation methods, depending on lipid compatibility and ion concentration requirements. For example, successful reconstitution of membrane transporters into proteo-GUVs has been achieved via electroformation in low-salt or salt-free buffers (see Suppl. Table S1). We anticipate that the experimental strategy presented here will serve as a valuable framework for defining the appropriate reconstitution conditions for other membrane transporters to preserve their activity in GUV systems.

## Materials and methods

### Materials

Phospholipids DPhPC, DOPC, DOPE, DOPG and DOTAP were obtained from Avanti Polar Lipids Inc. (Alabaster, AL, USA). Lecithin, obtained from Sigma-Aldrich (München, Germany), primarily contained phosphatidylcholine, phosphatidylethanolamine and several anionic phospholipids (Suppl. Figure S7). ATTO655-DOPE were purchased from ATTO-TEC GmbH (Siegen, Germany). Bio-Beads SM-2 Resin was obtained from BioRad Laboratories Inc. (Hercules, CA, USA). N-dodecyl-β-maltoside (DDM, > 99.5%) and n-octyl-β-D-glucoside (OG, > 99%) were obtained from Glycon (Luckenwalde, Germany). SNAP-Surface Alexa647 was obtained from New England BioLabs, Inc (MA, USA). The ionophores valinomycin and m-chlorophenylhydrazon (CCCP), the pH-sensitive dyes pyranine and 9-amino-6-chloro-2-methoxyacridine (ACMA), and all other chemicals and reagents were from Sigma-Aldrich (München, Germany), if not stated otherwise. ACMA was dissolved in dimethylsulfoxide. All solutions used for vesicles were filter-sterilized through a polyethersulfone membrane with a pore size of 0.2 μm (Filtropur, Sarstedt AG & Co. KG, Nümbrecht, Germany). Restriction endonucleases, T4 DNA ligase, and Q5 High-Fidelity DNA Polymerase were purchased from New England Biolabs.

### Plasmid construction

A plasmid based on the multicopy vector YEp-351^72^ was used for heterologous overexpression of a 73-amino acid C-terminal truncated AHA2 (AHA2∆73) in yeast, fused with a SNAP-tag and StrepII and hexahistidine (6× His) affinity tags at the N-terminus, under the control of the PMA1 promoter. To construct this SNAP-AHA2∆73 expression plasmid, the SNAP tag coding sequence was generated by PCR using primers *For.SNAP.INC* and *Rev.SNAP.INC* (Table 1), with the pENTR4-SNAPf(w878-1) plasmid (Addgene #29652) as the template. The PCR product, with 15 bp overhangs, was inserted into the XhoI/SpeI double-digested pMP1296^73^ plasmid using In-Fusion cloning (Takara Bio), generating a plasmid with compatible restriction sites for further cloning. The MRGSH6 and StrepII affinity tags, along with a TEV recognition site, were inserted upstream and downstream of the SNAP tag using primer sets HIS-STII and TEV-SITE (Table 1), resulting in the HIS-STII-SNAP-TEV plasmid. Finally, the AHA2∆73 coding sequence was amplified by PCR with primers For.AHA2.INC and Rev.AHA2.delta73.INC (Table 1), and cloned into the SacI/SpeI double-digested HIS-STII-SNAP-TEV plasmid using In-Fusion cloning.

**Table 1:** Nucleotide sequences of primers.

Primer	Sequence (5’→3’)
For.SNAP.INC	ATACCCCAGCCTCGAGAAGGGATCCGGTGACAAAGACTGCGAAATGAAG
Rev.SNAP.INC	TAGAACTAGAACTAGTGCCTGCAGGACCCAG
For.HIS-STII	TCGATAGCTAGTTAAAGAAAATCATTGAAAAGAATAACTCGAGTGATGAGAGGTTCTCATCACCATCACCATCACTGGTCTCATCCACAATTTGAAAAAG
Rev.HIS-STII	GATCCTTTTTCAAATTGTGGATGAGACCAGTGATGGTGATGGTGATGAGAACCTCTCATCACTCGAGTTATTCTTTTCAATGATTTTCTTTAACTAGCTA
For.TEV-SITE	GGTGGATCTGGTGGTTCTGAAAACTTGTATTTCCAAATGAGCTCTTAAA
Rev.TEV-SITE	CTAGTTTAAGAGCTCATTTGGAAATACAAGTTTTCAGAACCACCAGATCCACCTGCA
For.AHA2.INC	TTGTATTTCCAAATGTCGAGTCTCGAAGATATCAAGAACGAG
Rev.AHA2delta73.INC	TAGAACTAGAACTAGTTTACCATTGGGCCTCTCTCTCTTCT

### Expression and purification of AHA2

For expression of the SNAP-AHA2 fusion protein, the *Saccharomyces cerevisiae* strain RS-72 (*MATa ade1-100 his4-519 leu2-3*,*112*)^74^ was used. In this strain, the native yeast PM H^+^-ATPase PMA1 gene is placed under control of a galactose-dependent promoter. This strain can grow in galactose-containing media, but when cultured in a glucose-based medium, it relies on the presence of the constitutively expressed *Arabidopsis thaliana* H^+^-ATPase to complement the yeast H^+^-ATPase function. Cells were transformed with the SNAP-AHA2∆73 expression plasmid by the lithium acetate method^75^ and grown at 28–30 °C on rich synthetic SGAH plates (0.7% yeast nitrogen base, 2% galactose, 0.6% succinic acid, 2% agar with 0.004% adenine and 0.002% histidine)^76^. Yeast transformants were inoculated in 20 mL of SGAH medium and grown at 30 °C with 100 rpm in a Multitron shaker by INFORS HAT (Bottmingen, Switzerland). After 24 h, cells were inoculated into 200 mL of SGAH medium and grown for 24 h under the same conditions. Cells were inoculated for protein expression in 2 L YPDA (1% yeast extract, 2% bacto peptone, 2% D-glucose and 0.004% adenine) at 30 °C with shaking at 100 rpm. After 20–22 h of induction, cells were collected by centrifugation at 3,000 g for 5 min at 4 °C and washed in 50 mL of ice-cold water. The protein was purified according to previously published protocols^4,7,77^. The purified protein (1 − 10 mg/mL) in glycerol-containing buffer (50 mM MOPS-KOH, pH 7, 20% w/v glycerol, 50 mM KCl, 1 mM ethylenediaminetetraacetic acid, 1 mM dithiothreitol, 0.04% w/v DDM) was frozen in liquid nitrogen and stored at − 80 °C.

### LUV Preparation

Liposomes were prepared from dried lipid films, which were pre-mixed with specific lipid compositions required for different proteoliposome or GUV preparations. Briefly, 10 mg lipids dissolved in an organic solvent were mixed in small glass vials to achieve the desired molar ratio (Table 2) and dried under vacuum (250 mbar) for 3 h or ON. The lipid film was resuspended in the desired reconstitution buffer (Table 3) to a final concentration of 15 mg/mL by vortexing with a 5 mm glass bead for 10 min. Vesicles were extruded through two nucleopore polycarbonate membranes with a pore size of 0.2 μm using a mini-extruder (Avanti Polar Lipids), yielding LUVs.

**Table 2:** Lipid compositions used for the preparation of liposomes.

Lipid composition^1^	Molar ratio	Vesicles^1^	Application
DPhPC	1	PL	-
Lecithin	1	PL	Electroformation
Lecithin	1	PL	Gel-assisted swelling
DOPC: DOPG	7:3	GUV	Charge-mediated fusion
DOPC: DOTAP	9:1; 7:3	PL	Charge-mediated fusion
DOPC: DOPE: DOTAP	6:3:1	LUV	Charge-mediated fusion

^1^ Abbreviations: DPhPC, 1,2-diphytanoyl-*sn*-glycero-3-phosphocholine; DOPC, 1,2-dioleoyl-*sn*-glycero-3-phosphocholine; DOPG, 1,2-dioleoyl-*sn*-glycero-3-phospho-(1’-rac-glycerol); DOTAP, 2-dioleoyl-3-trimethylammonium-propane; DOPE, 1,2-diacyl-*sn*-glycero-3-phospho-L-serine; GUV, giant unilamellar vesicle; LUV, large unilamellar vesicle; PL, proteoliposome.

**Table 3:** Buffer compositions used for the preparation of liposomes.

Buffer	Composition
0 mM reconstitution buffer	10 mM MOPS-KOH, pH 7.0
15 mM reconstitution buffer	10 mM MOPS-KOH, pH 7.0, 15 mM K_2_SO_4_
35 mM reconstitution buffer	10 mM MOPS-KOH, pH 7.0, 35 mM K_2_SO_4_
50 mM reconstitution buffer	10 mM MOPS-KOH, pH 7.0, 50 mM K_2_SO_4_
300 mM ionic swelling buffer	10 mM MOPS-KOH pH 7.0, 50 mM K_2_SO_4_, 4 mM MgSO_4_, 100 µM pyranine, 132 mM sucrose

### Protein labelling

For SNAP labelling, 12–25 µg of purified AHA2 were diluted with labelling buffer (50 mM HEPES, pH 7.4, 100 mM KCl, 1 mM dithiothreitol, 0.04% w/v DDM) supplemented with 10 µM SNAP-Surface Alexa Fluor 488 or 647 (from a 1 mM stock in DMSO) and 0.5 g/L liposomes (final volume 43.87 µL) prior to incubation for 30 min on ice. Samples were analyzed by SDS-PAGE using 10% polyacrylamide gels and visualized on a ChemiDoc XRS Imaging System (Bio-Rad Laboratories GmbH, München, Germany) using the Image Lab™ software and pre-programmed option for Coomassie-stained gels and Alexa488/Alexa647 fluorophores, respectively.

### Proteoliposome preparation

Preformed LUVs (5 mM total lipid concentration) were reconstituted with 12–25 µg labelled or unlabeled AHA2 via a detergent destabilizing method using 25–30 mM OG and reconstitution buffer (Table 3). The protein/lipid/detergent mixture (200 µl) was subjected to gel filtration (Sephadex G-50 Fine, 3 mL packed in 1 cm diameter disposable syringes, Henry Schein, Langen, Germany) and 150–200 µl was recovered after centrifugation (180 x g, 8 min). The eluate was incubated for 30 min at room temperature with 100 mg of SM-2 Bio-Beads pre-washed in methanol followed by ddH_2_O (Bio-Rad Laboratories, Hercules, CA, USA) under overhead rotation to ensure detergent removal. To assess protein content relative to the specified controls, 10 µl of proteoliposomes were loaded onto a 10% SDS-PAGE gel and stained with Coomassie blue. The stained gels were analyzed with a ChemiDoc XRS Imaging System (Bio-Rad Laboratories GmbH, München, Germany) using Image Lab™ software and its pre-configured settings for Coomassie-stained gels.

### Monitoring AHA2 activity in proteoliposomes

Proton pumping by AHA2 into proteoliposomes was measured as the initial rate of ACMA fluorescence quenching^78^. Proteoliposomes (10 µL) were added to 1 mL ACMA buffer (10 mM MOPS-KOH, pH 7.0, 52.5 mM K_2_SO_4_, 2 mM ATP, 1 µM ACMA, and 62.5 nM valinomycin). Proton pumping was initiated by the addition of MgSO_4_ (3 mM final concentration), and the proton gradient dissipated by the addition of CCCP (5 µM final concentration, added from a 5 mM stock in ethanol). Fluorescence quenching was recorded over a period of 1,000 s with emission wavelength at 480 nm and excitation wavelength at 412 nm (Slit widths 5 nm, resolution, 0.1 s) at 23 °C using a fluorometer (PTI-Quantamaster 800, Horiba, Benzheim, Germany). Fluorescence traces were normalized to the intensity measured directly after the addition of MgSO_4_.

### Electroformation of GUVs or proteo-GUVs

GUVs were generated from proteoliposomes or liposomes in a chamber made of UV-cleaned glass slides coated with indium-tin-oxide (ITO; 28 × 1 mm, Nanion Technologies GmbH, München)^19,63,79^. Briefly, an O-ring was securely attached to the conductive side of one of the ITO-coated glass slides using vacuum sealing. Within the O-ring, 70 µL of proteoliposomes prepared in 50 mM reconstitution buffer were applied in 2 µL droplets and partially dehydrated under vacuum conditions (approximately 30–100 mbar) in a desiccator containing a saturated NaCl solution for 0.5–1 h at 4–6 °C. For the rehydration step, the inside of the O-ring was filled with 750 µL of a 300 mM ionic swelling buffer (Table 3). Subsequently, a chamber was created by sealing it with the second ITO glass slide. GUVs were formed overnight using the Nanion Vesicle Prep (Nanion Technologies GmbH, München) with the application of a sinusoidal voltage (0.35 V, 500 Hz).

### Gel-assisted formation of GUVs or proteo-GUVs

GUVs were generated from proteoliposomes or liposomes by swelling on top of a polyvinyl alcohol (PVA) film^35^. Briefly, 1 mL of 5% w/v PVA (Merck) solution in water was spread on a cleaned glass slide to form a thin film and the PVA film was dried at 60 °C for 30 min. Proteoliposomes prepared in 50 mM reconstitution buffer were applied as 2 µL droplets to a total volume of 50–60 µL on the PVA film and partially dehydrated under vacuum (approximately 30–100 mbar) in a desiccator containing a saturated NaCl solution for 0.5–1 h at 4–6 °C. Subsequently, an O-ring was glued down around the dried lipid film for chamber formation. The lipid film was hydrated in 650 µL of 300 mM ionic swelling buffer containing pyranine (Table 3) for 2–4 h.

### Charge-mediated reconstitution into GUVs

Negatively charged GUVs (50 µL, Table 2) prepared by electroformation using 300 mM ionic swelling buffer (Table 3) were mixed with positively charged proteoliposomes prepared using 50 mM reconstitution buffer (10 or 20 µL, Table 2) and incubated for 15 min at room temperature. For the fusion test, LUVs were prepared using 50 mM reconstitution buffer (Table 3) containing 1 mM pyranine. Afterwards, the free dye was removed using gel filtration (Sephadex G-50 Fine, 3 mL packed in 1 cm diameter disposable syringes).

### GUV imaging and analysis

Fluorescence microscopy and image acquisition were carried out using a Leica TCS SP8 confocal laser scanning microscope (Leitz, Wetzlar, Germany) equipped with a 63x/1.20 water objective. Images were acquired using 2048 × 2048 pixels, a 400 Hz unidirectional scanner, a pinhole of 100 μm (1 AU) with Leica hybrid photodetector (HyD SMD 2). The λex/λem used for imaging at laser intensity 5% were as follows: Alexa 488 488/500–600 nm, Alexa 647 650/655–700 nm, and pyranine 488/509–522 nm. GUVs were diluted 1:1 (v/v) in 300 mM microscopy buffer (10 mM MOPS-KOH pH 7, 50 mM K_2_SO_4_, 4 mM MgSO_4_, 62.5 nM valinomycin, 132 mM glucose). Before observation, GUVs were allowed to settle on a 24 × 50 mm cover glass slide for approximately 5 min. Proton pumping was initiated by the addition of 1 mM ATP from 0.5 M ATP-KOH pH 7 stock solution. Giant vesicles were observed under the microscope within 30 min, reducing the risk of unwanted vesicle collapse on the coverslip surface. ImageJ was used to analyze the fluorescence intensity of an area corresponding to the lumen of individual GUVs before and after adding ATP. Only unilamellar giant vesicles were used for analysis.

## Electronic supplementary material

Below is the link to the electronic supplementary material.

Supplementary Material 1

Supplementary Material 2

## Data Availability

The data in this study are all presented in the article. For further inquiries, please contact the corresponding author.
