# Supplementary material for: Functional reconstitution of plant plasma membrane H+-ATPase into giant unilamellar vesicles
Source: Sci Rep. 2025 Mar 12;15:8541. doi: 10.1038/s41598-025-92663-9 (PMC11903852; doi:10.1038/s41598-025-92663-9)
Supplement: Supplementary file 1 — Supplementary Material 1 [file 41598_2025_92663_MOESM1_ESM.pdf]

SUPPLEMENTARY INFORMATION II - SOURCE DATA

Functional reconstitution of plant plasma membrane H^+-ATPase into giant unilamellar vesicles

Huriye D. Uzun[1, 2], Ekaterina Malysenko[1], Bo H. Justesen[1] and Thomas Günther Pomorski[1, 2, *]

[1]Department of Molecular Biochemistry, Faculty of Chemistry and Biochemistry, Ruhr University Bochum, Bochum, Germany

[2]Department of Plant and Environmental Sciences, University of Copenhagen, Frederiksberg, Denmark

***Author for correspondence**

Thomas Günther Pomorski

Tel: +49 2343224430

Email address: thomas.guenther-pomorski@ruhr-uni-bochum.de

Figure 1B:

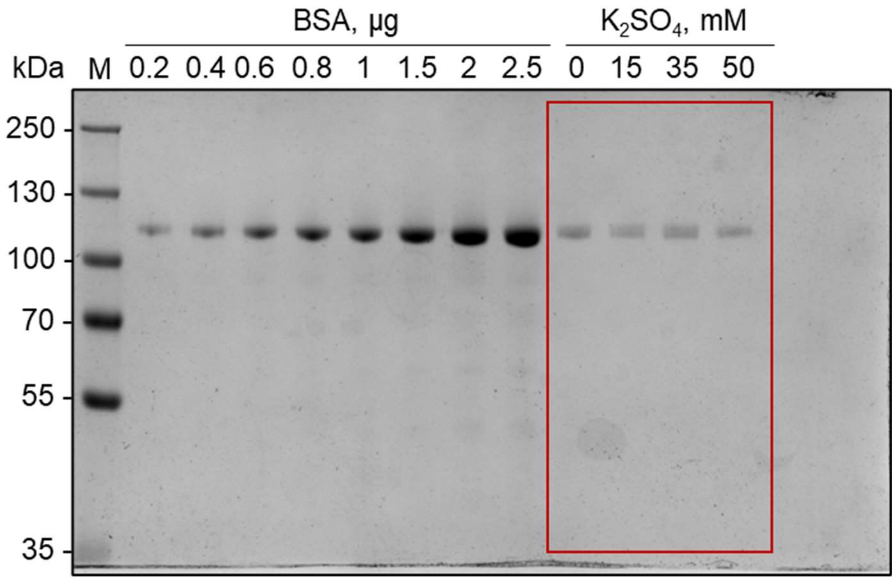

Coomassie Blue-stained SDS-PAGE gel of AHA2 reconstituted in proteoliposomes (PL) at the indicated ion concentrations and varying amounts of BSA. The gel runs against the PageRuler™ Plus Prestained Protein Ladder, 10 to 250 kDa.

Figure 2B:

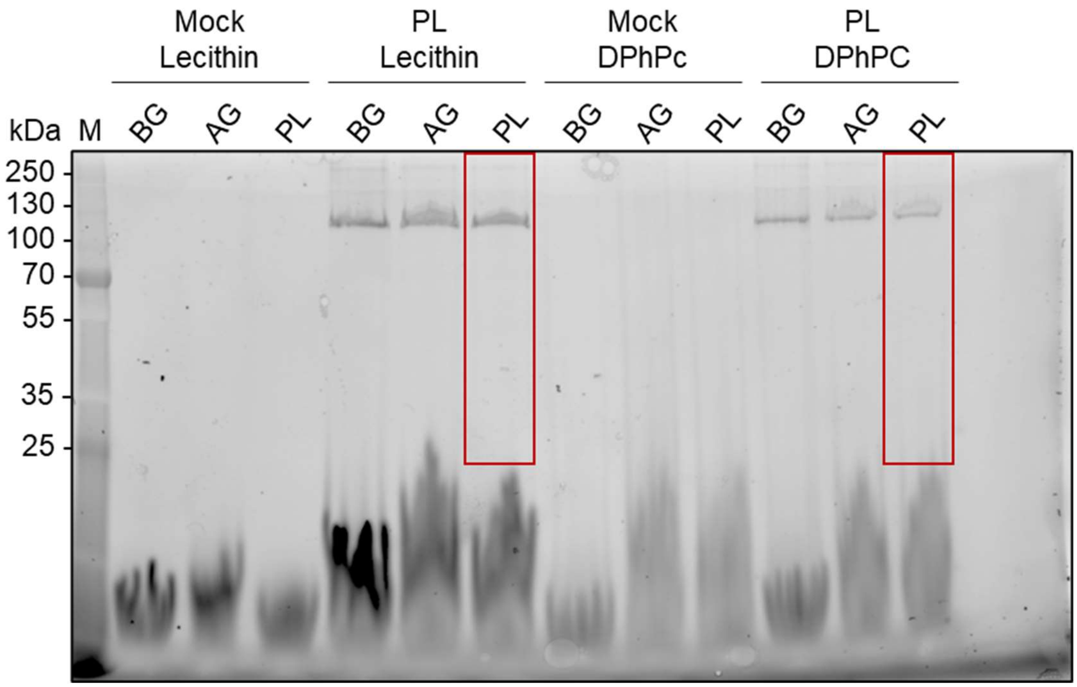

Coomassie Blue-stained SDS-PAGE gel of AHA2 reconstituted in proteoliposomes (PL) containing the indicated lipids and a protein-free control sample (Mock). During the reconstitution, samples were taken after mixing LUVs, H^+ pump, and detergent (BG), and the reconstitution mixture was run through a G50 column (AG), followed by incubation with Bio-Beads (PL) to remove the detergent. The gel runs against the PageRuler™ Plus Prestained Protein Ladder, 10 to 250 kDa.

Figure 2F:

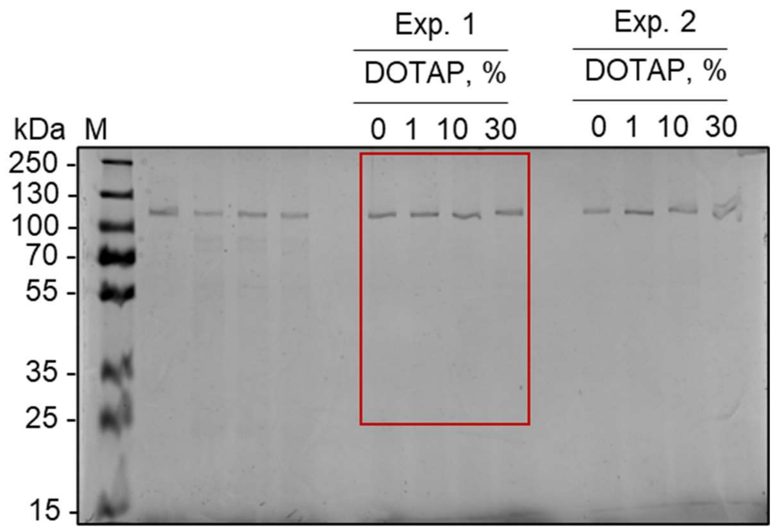

Coomassie Blue-stained SDS-PAGE gel of AHA2 reconstituted in proteoliposomes containing different molar ratios of DOTAP lipid. Two sets of experiments were loaded on the gel. The gel runs against the PageRuler™ Plus Prestained Protein Ladder, 10 to 250 kDa.

Suppl. Figure S1B:

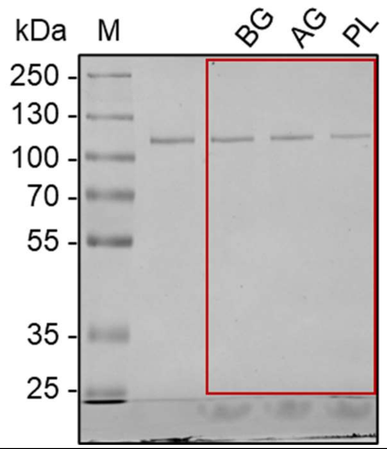

Suppl. Figure S2A:

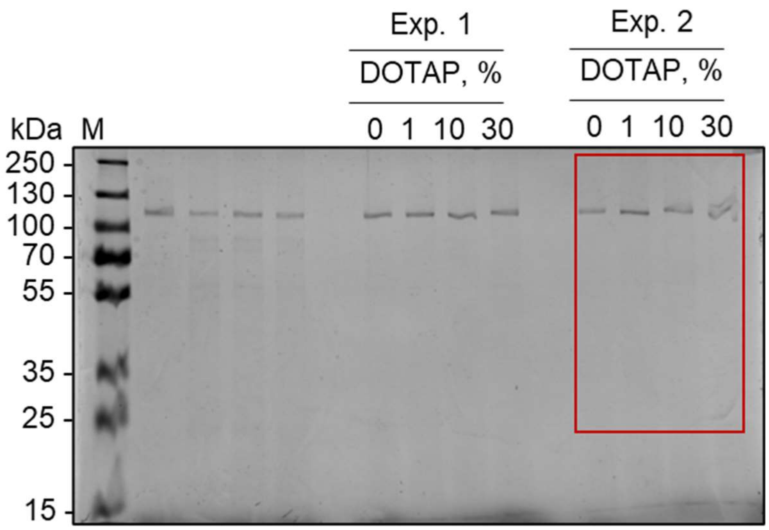

Coomassie Blue-stained SDS-PAGE gel of AHA2 reconstituted in proteoliposomes containing different molar ratios of DOTAP lipid. Two sets of experiments were loaded on the gel. The gel runs against the PageRuler™ Plus Prestained Protein Ladder, 10 to 250 kDa.

Suppl. Figure S3A:

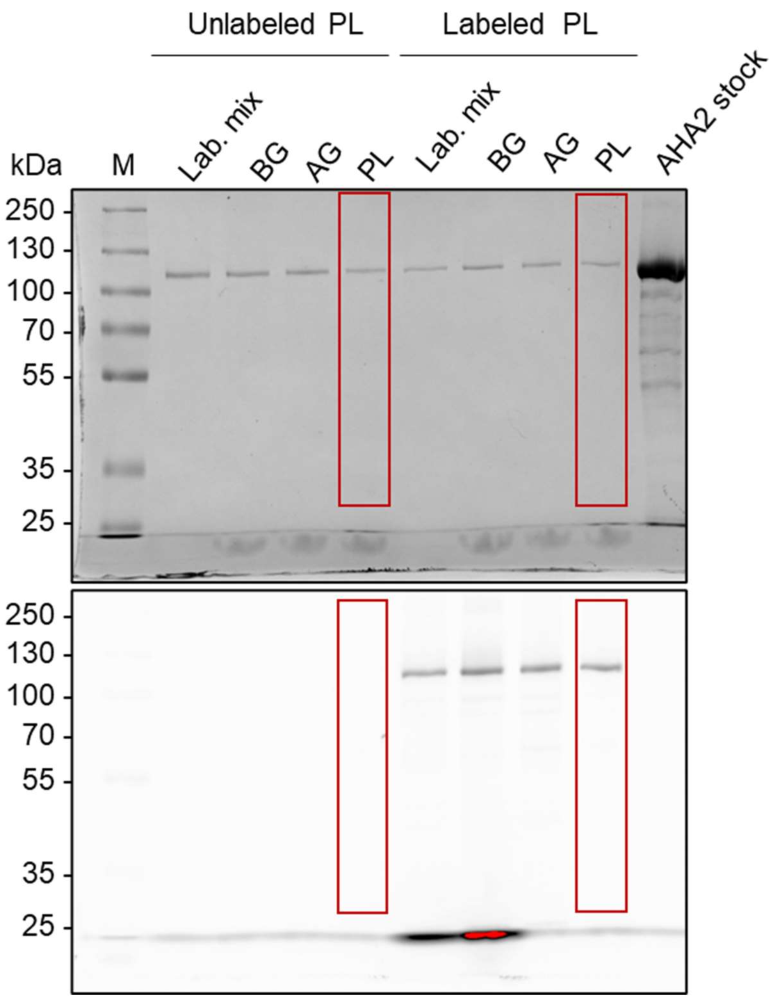

Upper panel: Coomassie Blue-stained SDS-PAGE gel of unlabeled and labeled AHA2 reconstituted in proteoliposomes (PL). During the reconstitution, samples were taken after mixing the H^+ pump with the label (lab. mix), then after mixing labeled or unlabeled H^+ pump, LUVs, and detergent (BG). The reconstitution mixture was run through a G50 column (AG), followed by incubation with Bio-Beads (PL) to remove the detergent. The gel runs against the PageRuler™ Plus Prestained Protein Ladder, 10 to 250 kDa.

Lower panel: Alexa647 scan of SDS-PAGE gel of unlabeled and labeled AHA2 reconstituted in PL.
