# Supplementary material for: Functional reconstitution of plant plasma membrane H+-ATPase into giant unilamellar vesicles
Source: Sci Rep. 2025 Mar 12;15:8541. doi: 10.1038/s41598-025-92663-9 (PMC11903852; doi:10.1038/s41598-025-92663-9)
Supplement: Supplementary file 2 — Supplementary Material 2 [file 41598_2025_92663_MOESM2_ESM.pdf]

SUPPLEMENTARY INFORMATION I

Functional reconstitution of plant plasma membrane H^+-ATPase into giant unilamellar vesicles

Huriye D. Uzun[1, 2], Ekaterina Malysenko[1], Bo H. Justesen[1] and Thomas Günther Pomorski[1, 2, *]

[1]Department of Molecular Biochemistry, Faculty of Chemistry and Biochemistry, Ruhr University Bochum, Bochum, Germany
[2]Department of Plant and Environmental Sciences, University of Copenhagen, Frederiksberg, Denmark

***Author for correspondence**

Thomas Günther Pomorski

Tel: +49 2343224430

Email address: thomas.guenther-pomorski@ruhr-uni-bochum.de

This file includes all supplementary information for the manuscript: Suppl. Table S1 and S2, Suppl. Figures S1-S7.

Suppl. Table S1: Overview of GUV-reconstituted ATP-driven membrane transporters, either based on GUV formation from proteoliposomes (PL) or direct detergent-mediated reconstitution.

Transporter	PL preparation		Proteo-GUVs /GUVs formation			Reference
	Lipid[1]	Buffer[1]	Lipid[1]	Buffer[1]	Approach[2]	
P-type ATPases						
Ca^{2+} ATPase (P2 ATPase)	EPC; DOPC; SOPC; EPC:EPA (9:1); DOPC:DOPS (9:1); DOPC:DOPE: DOPS (9:1:0; 8:1:1; 7:2:1); Asolectin; Brain lipids	2 mM MOPS-Tris, pH 7	-	1 mM MOPS-Tris pH 7, 0.1 mM $MgCl_2$/ 2 mM KCl, 100 mM sucrose	A (ITO, low *ƒ*)	[1]
Na^+/K^+ ATPase (P2 ATPase)	DOPC	30 mM histidine pH 7, 130 mM NaCl, 4 mM $MgCl_2$,	-	30 mM histidine pH 7, 30 mM NaCl, 2 mM $MgCl_2$, 200 mM sucrose	A (Pt wires, low *ƒ*)	[2]
Na^+/K^+ ATPase (P2 ATPase)	DOPC:Chol (60:40); DOPC:DOPS: Chol (40:20:40)	30 mM histidine pH 7, 130 mM NaCl, 4 mM $MgCl_2$	-	30 mM histidine pH 7, 30 mM NaCl, 2 mM $MgCl_2$, 200 mM sucrose	A (Pt wires, low *ƒ*)	[3]
PcoB, Heavy metal ATPase (P1 ATPase)	DPhPC	-	-	20 mM MOPS-NaOH pH 7.4, 150 mM NaCl/Na_2SO_4, 15 mM KCl/K_2SO_4, 50 µM FluoZin-3, 5 µM valinomycin, 100 mM raffinose	B (Agarose)	[4]
SERCA Ca^{2+} ATPase (P2 ATPase)	DOPC:DOPE (8:2)	-	DOPC:EDOPC (100-90:0–10%; w/w)	1 mM MOPS-Tris pH 7.0, 100 mM sucrose	C (ITO, low *ƒ*)	[5]
CopA, Cu-ATPases (P1 ATPase)	-	-	POPC and DDM	350 mM sucrose	E (A, ITO; low *ƒ*)	[6]
ABC transporters						
Hamster P-glycoprotein	POPC; POPC:Chol (68.8:30; 61.8:37)	20 mM HEPES pH 7.5, 100 mM NaCl, 5 mM $MgCl_2$	-	235 mM sucrose	A (ITO, low *ƒ*)	[7]
Human P-glycoprotein	PC:PS:Chol (60:17.5:10:12.5; w:w)	50 mM Tris–HCl, pH 7.4, 1.25% octylglucoside	-	50 mM Tris–HCl pH 7, 0.1 mM DTT, 190 mM sucrose	B (Agarose)	[8]
TmrAB	POPC:POPG: POPE (40:30:29 mol%); E. coli polar lipids:DOPC (70:30 mol%)	20 mM HEPES-NaOH pH 7.5, 150 mM NaCl	-	200 mM Sucrose	B (PVA gel)	[9]
BR	DOPC:Chol (3.5:1.5)	20 mM PIPES pH 7.2, 110 mM K_2SO_4	DOPC:DOPE:SAINT-2, (10:3:0.65; 10:3:1.3)	20 mM PIPES, pH 7.2, 0.2 mM pyranine, 0.1 µM valinomycin	D (ITO, low *ƒ*)	[10]

BmrC/BmrD, bacterial	E. coli lipids	50 mM Tris-HCl pH 8, 150 mM KCl, 25 mM NaCl, 4% (v/v) glycerol, 1 mM β-mercaptoethanol	DPhPC; DOPC:DOPE (1:1); DOPC:Sph:Chol (33:33:3), 75 μM DOTM	400 mM sucrose	E (A, ITO; low *f*)	[11]
BR, Light-driven H^+ pump	-	-	EPC:EPA (9:1); EPC; DPhPC; 75 μM DOTM	400 mM sucrose	E (A, ITO; low *f*)	[11]
F-type ATPases						
F1F0-ATP synthase	E. coli lipid extract	50 mM Tris-HCl pH 8, 3 mM KCl	E. coli extract	0.15 mM pyranine, 200 mM sucrose pH 6	A (ITO, high f)	[12]
F1F0-ATP synthase	-	-	E. coli extract, DDM	0.15 mM pyranine, 200 mM sucrose pH 6	E (A, ITO; high *f*)	[12]
F1F0-ATP synthase	Soybean PC	High ionic strength	-	10 mM HEPES-KOH pH 7.5, 50 mM KCl, 2 mM $MgSO_4$, 1 mM ATP	B (glass slide)	[13]
F1F0-ATP synthase	PE:PC:Chol (35:35:30)	10 mM HEPES, pH 7.3, 120 mM KCl, 50 mM NaCl	PC	10 mM HEPES pH 7.3, 120 mM KCl, 8 mM NaCl, 0.5 mM EGTA	B (glass slide)	[14]
F1F0-ATP synthase	DOPC:DOTAP (7 : 3)	20 mM Hepes, pH 7.5, 2.5 mM $MgCl_2$, 25 g/l sucrose	DOPC:DOPG (7:3)	400mM sucrose	C (ITO, low high *f*)	[15]

[1]Abbreviations used for lipids and buffers: Chol, cholesterol; DDM, n-Dodecyl-β-Maltoside; DOPC, 1,2-dioleoyl-*sn*-glycero-3-phosphocholine; DOPE, 1,2-diacyl-*sn*-glycero-3-(phospho-l-serine); DOPS, 1,2-diacyl-*sn*-glycero-3-(phospho-l-serine); DOTM, n-dodecyl-β-D-thiomaltopyranoside; DPhPC, 1,2-diphytanoyl-*sn*-glycero-3-phosphocholine; DTT, 1,4-Dithio-D-threitol; E. coli, Escherichia coli; EGTA, ethylene glycol-bis(β-aminoethyl ether)-N,N,N′,N′-tetraacetic acid; EPA, egg l-a-phosphatidic acid; EPC/EDOPC, egg l-α-phosphatidylcholine; HEPES, (4-(2-hydroxyethyl)-1-piperazineethanesulfonic acid); MOPS, 3-(N-morpholino)propanesulfonic acid; PC, phosphatidylcholine; PE, phosphatidylethanolamine; PIPES, piperazinediethanesulfonic acid; POPC, 1-palmitoyl-2-oleoyl-*sn*-glycero-3-phosphocholine; POPE, 1-palmitoyl-2-oleoyl-*sn*-glycero-3-phosphatidylethanolamine; POPG, 1-palmitoyl-2-oleoyl-*sn*-glycero-3-phosphatidylglycerol; PS, phosphatidylserine; SAINT-2, N-methyl-4-(dioleyl)methylpyridinium chloride; SOPC, 1-stearolyl-2-oleoyl-*sn*-glycero-3-phosphocholine; Sph, sphingomyelin; Tris, tris(hydroxymethyl)aminomethane.

[2]Abbreviations used for approaches: (A) Electroformation: Proteoliposomes, or lipid/protein solutions are dehydrated or partially dehydrated on indium tin oxide (ITO) or platinum wires (Pt) and afterwards rehydrated in the presence of an AC electrical field (high or low frequency (f)). (B) Gel-assisted swelling: Proteoliposomes or lipid/protein solutions are dehydrated or partially dehydrated on a glass slide or on a gel-coated (agarose or poly(vinyl alcohol) (PVA)) glass slide and afterwards rehydrated spontaneously. (C) Charge-mediated fusion: Preformed charged GUVs and oppositely charged proteoliposomes are fused using their charged properties (D) Peptide-induced fusion: proteoliposomes, to which the small fusogenic peptide WAE has been covalently attached, are fused with GUV. (E) Direct (detergent-mediated) reconstitution: Purified and detergent-solubilized protein is added to preformed GUVs prepared from lipids or lipids and detergent.

Suppl. Table S2: Summary of conditions and results for proteoliposome and proteo-GUV preparation.

Sample[1]	Lipids[2]	Buffer[3]	Generation of PL or proteo-GUV	H^+-pumping activity	Figure
Testing different PL conditions	Lecithin	10 mM MOPS-KOH, pH 7.0	+	-	Fig. 1
		10 mM MOPS-KOH, pH 7.0, 15 mM K_2SO_4	+	-	Fig. 1
		10 mM MOPS-KOH, pH 7.0, 35 mM K_2SO_4	+	+	Fig. 1
		10 mM MOPS-KOH, pH 7.0, 50 mM K_2SO_4	+	+++	Fig. 1
	DPhPC	10 mM MOPS-KOH, pH 7.0, 50 mM K_2SO_4	-	-	Fig. 2 A-D
	DOPC		+	+	Fig. 2 E-H & Suppl. Fig. S2
	DOPC:DOTAP (9.9:0.1)		+	+	Fig. 2 E-H & Suppl. Fig-S2
	DOPC:DOTAP (9:1)		+	+	Fig. 2 E-H & Suppl. Fig. S2
	DOPC:DOTAP (7:3)		+	-	Fig. 2 E-H & Suppl. Fig. S2
Test the labeling of AHA2	Lecithin	10 mM MOPS-KOH, pH 7.0, 50 mM K_2SO_4	+	+++	Suppl. Fig. S3
Generation of proteo-GUV –electrofomation	Lecithin	10 mM MOPS pH 7.0, 50 mM K_2SO_4, 4 mM $MgSO_4$, 132 mM sucrose	+	-	Fig. 3
Generation of proteo-GUV –gel-assisted swelling		10 mM MOPS pH 7.0, 50 mM K_2SO_4, 4 mM $MgSO_4$, 100 µM pyranine, 132 mM sucrose	++	++	Fig. 3, Fig. 4 & Suppl. Fig. S6
Generation of proteo-GUV –charge-mediated fusion	DOPC:DOPG (7:3) GUVs with DOPC:DOTAP (9:1) PL	10 mM MOPS pH 7.0, 50 mM K_2SO_4, 4 mM $MgSO_4$, 132 mM sucrose	-	-	Fig. 3 & Suppl. Fig. S4
	DOPC:DOPG (7:3) GUVs with DOPC:DOTAP (7:3) LUVs		-	-	Fig. 3 & Suppl. Fig. S4
	DOPC:DOPG (7:3) GUVs with DOPC:DOPE:DOTAP (6:3:1) LUVs		-	-	Fig. 3 & Suppl. Fig. S4

[1]Abbreviations used for samples; proteo-GUV, protein containing giant unilamellar vesicle; PL, proteoliposomes.

[2]Abbreviations used for lipids: DOPC, 1,2-dioleoyl-*sn*-glycero-3-phosphocholine; DOPE, 1,2-diacyl-*sn*-glycero-3-(phospho-l-serine); DOPG, 1,2-dioleoyl-sn-glycero-3-phospho-(1'-rac-glycerol); DOTAP, 2-dioleoyl-3-trimethylammonium-propane; DPhPC, 1,2-diphytanoyl-*sn*-glycero-3-phosphocholine.

[3]Abbreviations used buffer: MOPS, 3-(N-morpholino)propanesulfonic acid.

A)

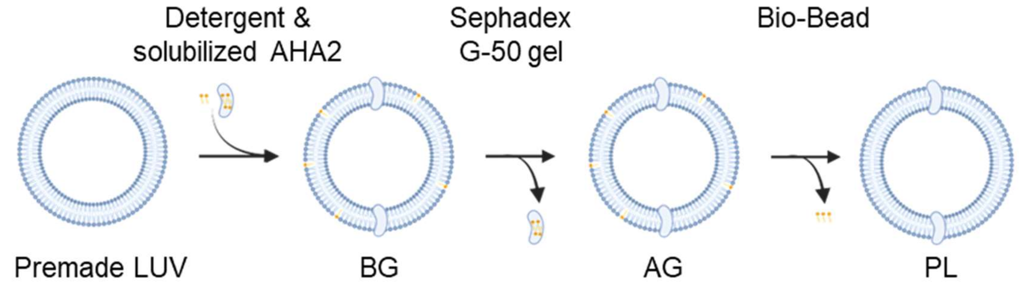

B)

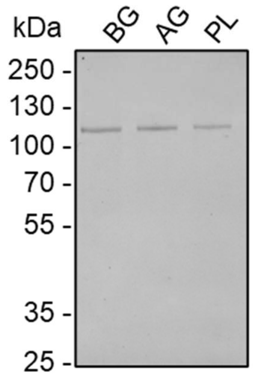

C)

Sample	AHA2 recovery, %
BG	100.0
AG	90.1 ± 9.4
PL	62.8 +11.6

Suppl. Figure S1: Recovery of AHA2 during the reconstitution into liposomes. A) Schematic diagram illustrating the reconstitution of AHA2. Preformed liposomes (LUV) are detergent-destabilized and mixed with the detergent-solubilized H^+-ATPase AHA2 (BG). Subsequent removal of the detergent by Sephadex G-50 gel filtration (AG) and Bio-Bead treatment result in the formation of sealed proteoliposomes (PL). B) Representative Coomassie Brilliant Blue-stained SDS-PAGE of samples collected at the indicated stages of the reconstitution process. Images were acquired using the GelDoc system (Bio-Rad). The bands observed in all three lanes correspond to the expected molecular weight of truncated AHA2 (119 kDa), confirming its presence throughout the reconstitution process. C) Protein recovery of AHA2 was assessed via band intensity using Image Lab software, showing 62.8 ± 11.6% (n=2) incorporation into vesicles.

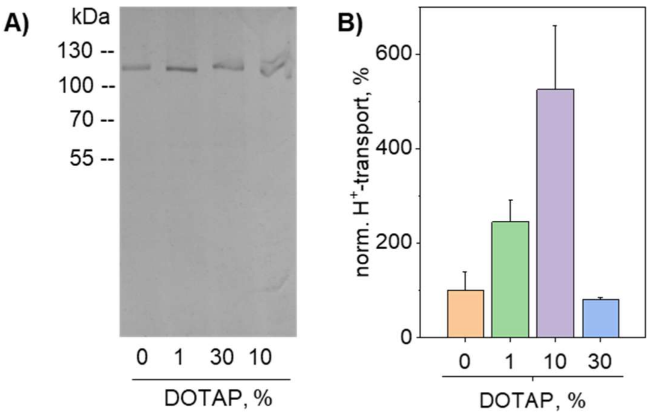

Suppl. Figure S2: AHA2 activity upon reconstitution in LUVs containing DOTAP lipid. Data shown corresponds to a second independent reconstitution preparation. A) Coomassie Blue-stained SDS-PAGE gel of AHA2 reconstituted in proteoliposomes containing various DOTAP concentration. B) Proton transport activity of AHA2 was determined as initial rates of the fluorescence quenching of the pH sensor ACMA, normalized to liposomal protein content, and expressed relative to the control (0% DOTAP). In panel B, data represents one reconstitution with three measurements, shown as mean ± S.D. A value of 100% corresponds to $7 \pm 3 \times 10^{-6}$ %/s.

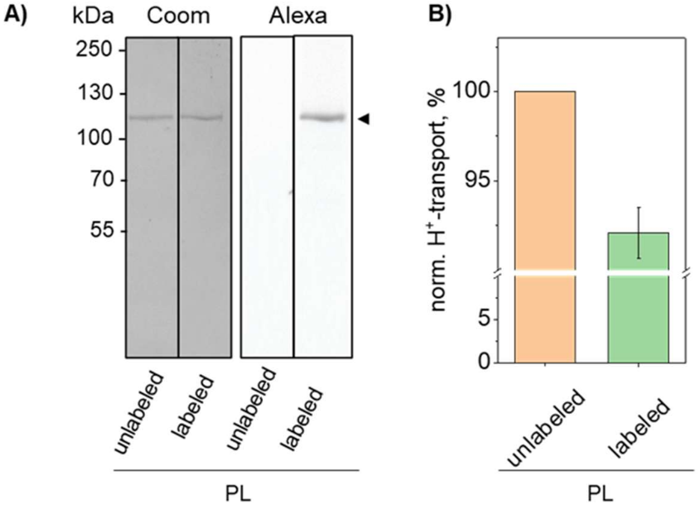

Suppl. Figure S3: Functional characterization of Alexa647-labeled and unlabeled AHA2 reconstituted into large unilamellar vesicles. A) Alexa647 fluorescence and Coomassie-stained SDS-PAGE confirm the incorporation of labeled and unlabeled AHA2 into proteoliposomes (PL). B) Proton transport activity of AHA2 was assessed by measuring the initial rate of fluorescence quenching of the ΔpH-sensitive dye ACMA, normalized to liposomal protein content, and expressed relative to unlabeled AHA2 proteoliposomes. Data are the means ± range of two independent experiments. A value of 100% corresponds to $1.75 \pm 1.15 \times 10^{-5}$ %/s.

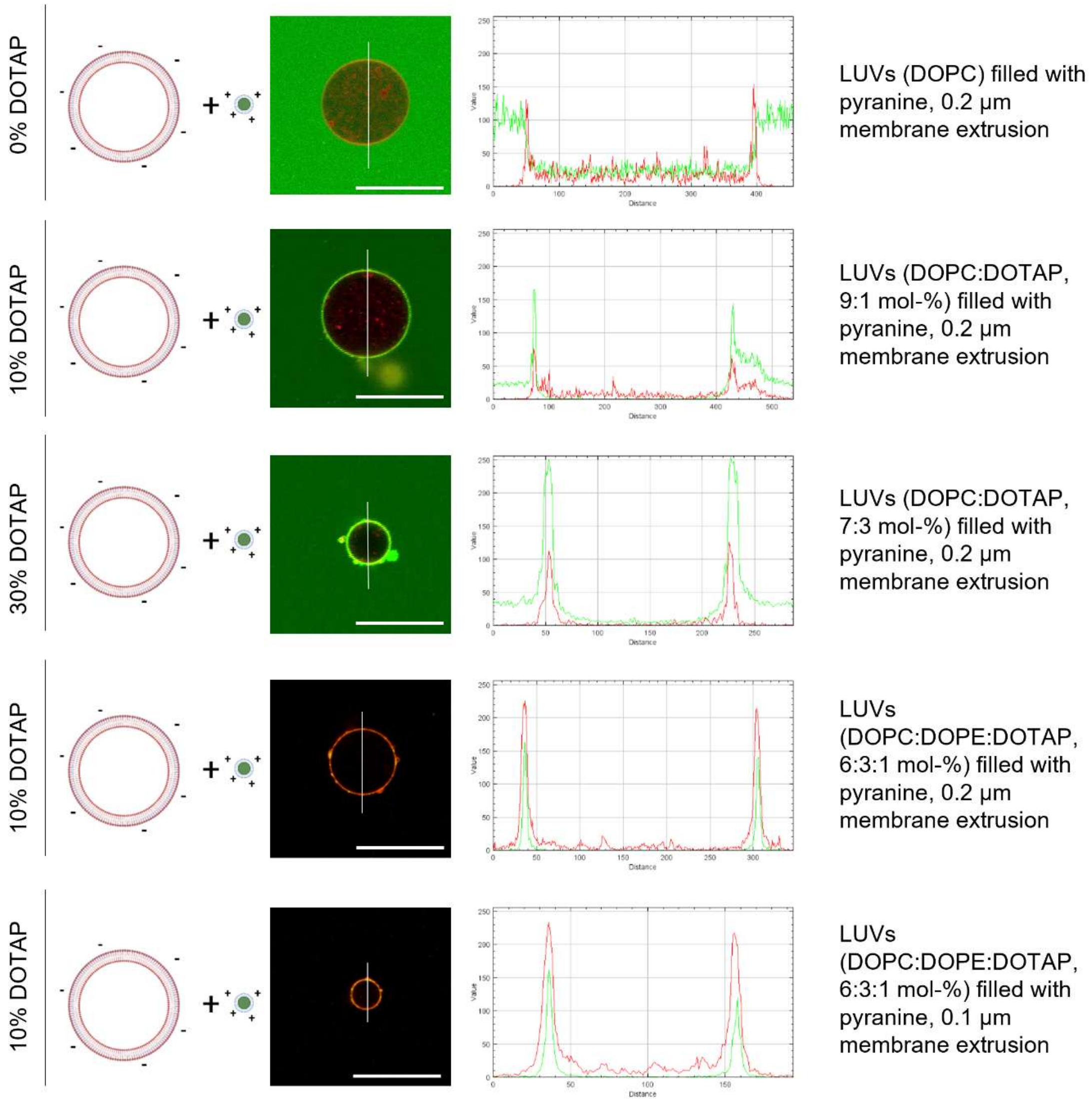

Suppl. Figure S4: Probing charge-mediated fusion between oppositely charged LUVs and GUVs. GUVs were prepared using the lipid composition DOPC:DOPG (molar ratio 7:3) with added 0.2 mol% ATTO655-DOPE by the electroformation method. Pyranine-loaded LUVs with the indicated lipid composition were prepared using the extrusion method. In the last two conditions, pyranine-loaded LUVs were passed through a Sephadex G-50 column to remove external pyranine, resulting in low background fluorescence. LUVs and GUVs were then incubated together for 15 min at room temperature or 37°C. The resulting LUV-GUV mixtures were observed using confocal microscopy in microscopy buffer. Overlay images of both the red (ATTO655-DOPE) and the green (pyranine) channels were analyzed for fluorophore intensity peaks. For colocalization analysis of the reporter dyes, a straight line was drawn over a single vesicle, and the fluorescence intensity was plotted against the distance on the line in a diagram. Scale Bars, 20 µm.

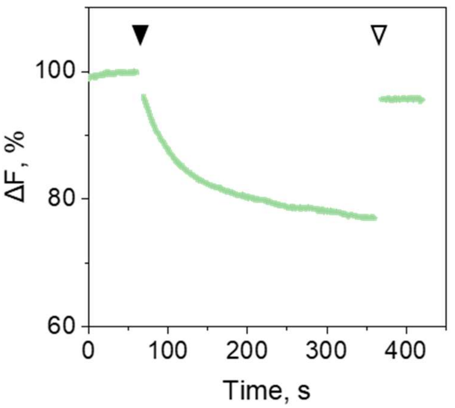

Suppl. Figure S5: AHA2 activity upon reconstitution into pyranine-filled large unilamellar vesicles. Lecithin was dissolved in reconstitution buffer containing 25 mM pyranine and extruded through two nucleopore polycarbonate membranes with a pore size of 0.2 µm using a mini-extruder (Avanti Polar Lipids), yielding LUVs. AHA2 was reconstituted into these preformed LUVs using the detergent destabilization method, as described in under "Materials and methods". Proton transport into pyranine-filled vesicles reconstituted with AHA2 was measured at 23°C using a fluorometer (PTI-Quantamaster 800, Horiba, Benzheim, Germany; slit widths 5 nm, resolution 0.1 s), in a buffer containing 10 mM MOPS-KOH pH 7.0, 50 mM K_2SO_4, 3 mM $MgSO_4$, and 62.5 nM valinomycin, with excitation and emission wavelengths set to 462 nm and 512 nm, respectively. ATP-stimulated proton pumping was initiated by adding 2 mM ATP (filled arrowhead), leading to a decrease in pyranine fluorescence due to vesicular acidification. The proton gradient was subsequently dissipated by adding the protonophore m-chlorophenylhydrazone (CCCP), resulting in fluorescence recovery. The fluorescence signal was normalized to the baseline signal recorded before ATP addition.

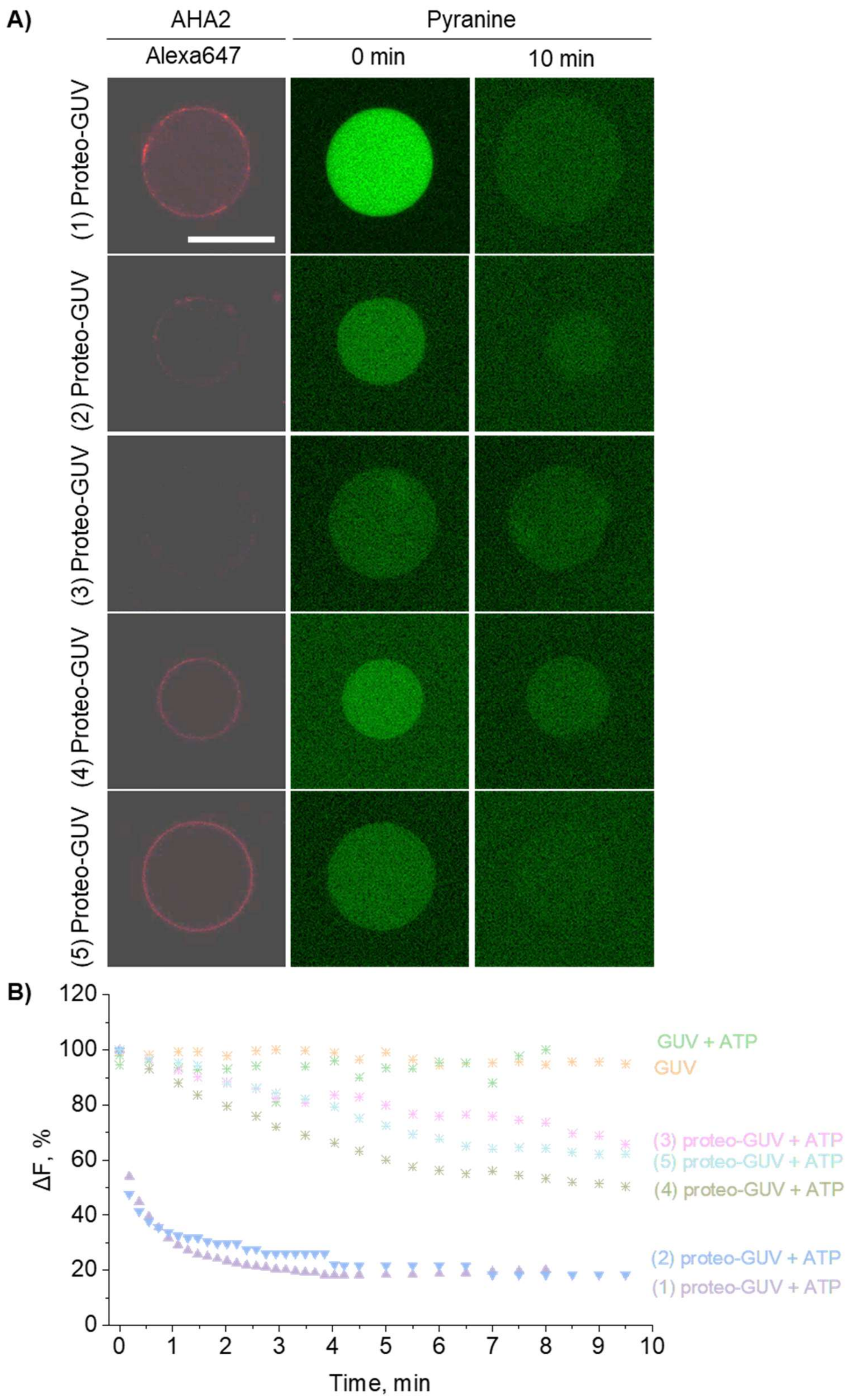

Suppl. Figure S6: AHA2 activity upon reconstitution into proteo-GUVs. A) Confocal images of proteo-GUVs formed via the gel-assisted swelling method in the presence of pyranine. Vesicles were analyzed by fluorescence microscopy before and after the addition of ATP. Scale bar, 20 μm. B) Pyranine fluorescence of proteo-GUVs and GUVs (control) measured as a function of time, normalized to the maximum fluorescence value. The data represent different proteo-GUV preparations and H^+ measurements and include traces from Figure 4 of the main manuscript. Note that proton pump activity for the individuel GUVs is influenced by several factors, including protein amount, membrane orientation, and the size of the GUVs.

A)

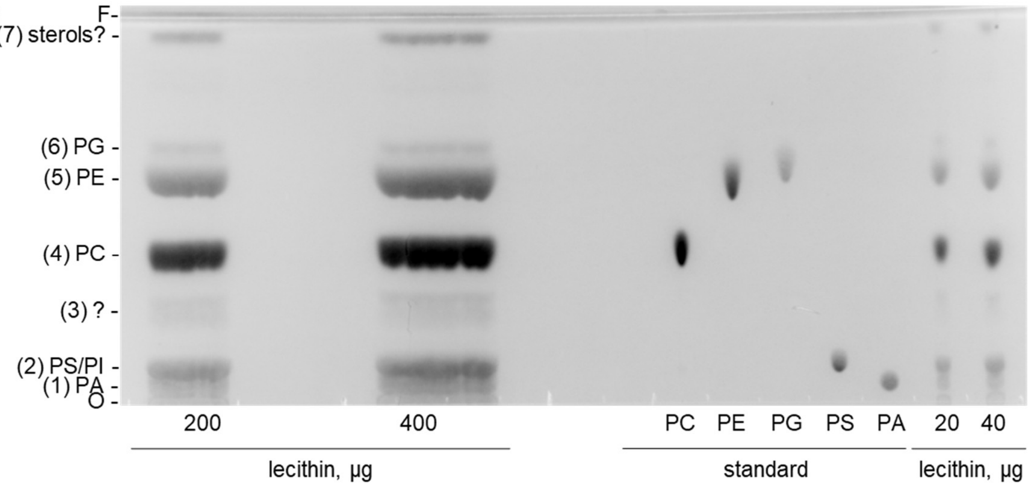

B)

numbers	lipids	average, %
1	PA	5.2 ± 2.1
2	PS/PI	13.0 ± 1.6
3	?	2.4 ± 0.3
4	PC	43.8 ± 3.4
5	PE	18.9 ± 1.7
6	PG	1.8 ± 0.7
7	sterols	3.9 ± 0.6

Suppl. Figure S7 : Lecithin composition analysis by thin-layer chromatography (TLC). A) Lipid separation was performed using a chloroform:methanol:ammonium hydroxide solvent system (63:35:5, v/v/v). Two lecithin concentrations were analyzed alongside standard lipid spots, including 1,2-dioleoyl-*sn*-glycero-3-phosphocholine (PC), 1,2-dioleoyl-*sn*-glycero-3-phosphoethanolamine (PE), 1,2-dioleoyl-*sn*-glycero-3-phospho-(1'-rac-glycerol) (PG), 1,2-dioleoyl-*sn*-glycero-3-phospho-L-serine (PS), and 1,2-dioleoyl-*sn*-glycero-3-phosphate (PA), on the same TLC plate. Note that PS and phosphatidylinositol (PI) cannot be separated under the TLC conditions used. Chromatograms were dried completely before visualization, stained with 0.005% primuline in acetone:water (8:2, v/v), and imaged under long-wave UV light using the Bio-Rad ChemiDoc XRS Imaging System. O, origin; F, front B) The lipid composition of lecithin was determined by calculating the percentage of each band's volume relative to the total volume of the lane. Four lanes (20, 40, 200, 400 µg) were analysed using the Bio-Rad ChemiDoc XRS Imaging System. Values are mean ± S.D.

References

1. Girard, P. *et al.* A new method for the reconstitution of membrane proteins into giant unilamellar vesicles. *Biophysical journal* **87,** 419–429 (2004).
2. Bhatia, T., Cornelius, F., Mouritsen, O. G. & Ipsen, J. H. Reconstitution of transmembrane protein Na+,K+-ATPase in giant unilamellar vesicles of lipid mixtures involving PSM, DOPC, DPPC and cholesterol at physiological buffer and temperature conditions. *Protocol Exchange*; 10.1038/protex.2016.010 (2016).
3. Bouvrais, H., Cornelius, F., Ipsen, J. H. & Mouritsen, O. G. Intrinsic reaction-cycle time scale of Na+,K+-ATPase manifests itself in the lipid-protein interactions of nonequilibrium membranes. *Proceedings of the National Academy of Sciences of the United States of America* **109,** 18442–18446 (2012).
4. Górecki, K. *et al.* Microfluidic-Derived Detection of Protein-Facilitated Copper Flux Across Lipid Membranes. *Analytical chemistry* **94,** 11831–11837 (2022).
5. Bian, T. *et al.* Direct detection of SERCA calcium transport and small-molecule inhibition in giant unilamellar vesicles. *Biochemical and biophysical research communications* **481,** 206–211 (2016).
6. Wijekoon, C. J. K. *et al.* Copper ATPase CopA from Escherichia coli: Quantitative Correlation between ATPase Activity and Vectorial Copper Transport. *Journal of the American Chemical Society* **139,** 4266–4269 (2017).
7. Park, S. & Majd, S. Reconstitution and functional studies of hamster P-glycoprotein in giant liposomes. *PloS one* **13,** e0199279 (2018).
8. Horger, K. S. *et al.* Hydrogel-assisted functional reconstitution of human P-glycoprotein (ABCB1) in giant liposomes. *Biochimica et biophysica acta* **1848,** 643–653 (2015).
9. Diederichs, T. & Tampé, R. Single Cell-like Systems Reveal Active Unidirectional and Light-Controlled Transport by Nanomachineries. *ACS nano* **15,** 6747–6755 (2021).
10. Kahya, N., Pécheur, E. I., Boeij, W. P. de, Wiersma, D. A. & Hoekstra, D. Reconstitution of membrane proteins into giant unilamellar vesicles via peptide-induced fusion. *Biophysical journal* **81,** 1464–1474 (2001).
11. Dezi, M., Di Cicco, A., Bassereau, P. & Lévy, D. Detergent-mediated incorporation of transmembrane proteins in giant unilamellar vesicles with controlled physiological contents. *Proceedings of the National Academy of Sciences of the United States of America* **110,** 7276–7281 (2013).
12. Almendro-Vedia, V. G. *et al.* Nonequilibrium fluctuations of lipid membranes by the rotating motor protein F1F0-ATP synthase. *Proceedings of the National Academy of Sciences of the United States of America* **114,** 11291–11296 (2017).
13. Onoue, Y. *et al.* A giant liposome for single-molecule observation of conformational changes in membrane proteins. *Biochimica et biophysica acta* **1788,** 1332–1340 (2009).
14. Mnatsakanyan, N. *et al.* A mitochondrial megachannel resides in monomeric F1FO ATP synthase. *Nature communications* **10,** 5823 (2019).

15. Biner, O., Schick, T., Müller, Y. & Ballmoos, C. von. Delivery of membrane proteins into small and giant unilamellar vesicles by charge-mediated fusion. *FEBS letters* **590,** 2051–2062 (2016).
